# The analgesic effect of music on cold pressor pain responses: The influence of anxiety and attitude toward pain

**DOI:** 10.1371/journal.pone.0201897

**Published:** 2018-08-06

**Authors:** Suvin Choi, Sang-Gue Park, Hyung-Hwan Lee

**Affiliations:** 1 Graduate School of Public Health & Institute of Health and Environment, Seoul National University, Seoul, Korea; 2 Da Vinci College of General Education, Chung-Ang University, Seoul, Korea; 3 Department of Applied Statistics, Chung-Ang University, Seoul, Korea; 4 Department of Traditional Music, Chung-Ang University, Anseong, Korea; Universitat Wien, AUSTRIA

## Abstract

**Objective:**

The analgesic effect of music has been recognized for pain relief, but individual differences and adjuvant methods are poorly understood. This study employed a cold-pressor task (CPT) to observe the effects of music (without considering personal preferences) on pain experience and how this is affected by individuals’ general (and pain-specific) anxiety symptomology.

**Methods:**

Fifty participants were each presented with three conditions (randomized into different orders): music-listening, news-listening, and no sound (control). Pain responses, including pain tolerance time (PT), pain intensity (PI), and pain unpleasantness (PU), were assessed using CPT and compared with a 3x3 crossover design. Participants also completed the anxiety sensitivity index (ASI-16) and pain anxiety symptom scale (PASS-20).

**Results:**

CPT pain responses during the music intervention were significantly different from responses during the news intervention and control conditions, respectively. Among participants with normal anxiety levels, pain responses during the music condition differed significantly from the news and control groups; this was not the case for the anxiety risk group. Pain responses during the music condition for those with normal levels of pain-specific anxiety differed significantly from the control, but this was not the case for the risk group.

**Conclusions:**

Music appears to influence diminished pain responses relative to the absence of an intervention. However, this was not the case when individuals listened to news stories. These effects were more robust for individuals experiencing normal levels of general and pain-specific anxiety. Thus, music (even outside one’s own preferences) was an effective adjuvant method for managing pain, especially among those without significant anxiety symptomology.

## 1. Introduction

Pain is an uncomfortable sensory and emotional experience, which is typically caused by actual (or potential) tissue injury or mental stress [1, 2]. When not effectively treated or relieved, pain can negatively impact quality of life. Pain research has not only focused on understanding pain as a phenomenon but also on how pain is recognized and tolerated [3–5]. However, pain management without the aid of pharmaceutical treatments is still a difficult issue for healthcare professionals and patients [6].

Music has been investigated as a potentially viable non-pharmacological treatment for alleviating pain [7–9]. Additionally, listening to music along with taking oral sedatives during cataract surgery reduced anxiety and enhanced comfort [10], diminishing the experience of pain during the surgery [11]. Recent research has revealed that music plays a critical role in dispersing harmful stimulants in order to transform cognitive faculties and emotions as an “audio analgesic” [9, 12–14]. Several studies have also examined music as an adjuvant method and observed that music helps alleviate painful conditions. Most studies typically considered participants’ music preferences to act as a key component for effectively decreasing pain [15–18].

Nevertheless, controversies and limitations remain, particularly regarding control over music selection, methodological and experimental shortcomings [19–21], and the role of individual psychological states. In the present study, we aimed to determine whether selecting music (without taking into account individual preferences) would still be effective at relieving pain. Additionally, we examined whether any audio analgesic effects are specific to music or any other auditory stimulus. Thus, we created three experimental conditions: researcher-chosen music, weather news, and a no-sound control condition. We specifically selected music and news stimuli that would be well recognized but not overly preferred. Music that minimized familiarity and engagement among the participants was chosen. This same approach was applied when selecting news clips as the alternative sound medium [13, 22].

The second issue addressed in the present study was to choose a research design suitable for comparing pain responses based on individual participant variability. Crossover designs are useful for avoiding the influence of confounding factors while enhancing efficiency, controlling for individual differences by assigning each participant to all treatment conditions [23, 24]. A previous study by Choi et al. [11] utilized a 2x2 crossover design to show that music has an analgesic effect during cataract surgery. Following a similar design, we employed a 3x3 crossover design to compare music-listening, news-listening, and no-sound control conditions in relation to cold pressor pain (CPT) responses.

Finally, previous studies have observed relationships between pain and anxiety [25]; thus, listening to music could modulate stress-induced anxiety and/or pain-specific anxiety levels. Considering prior studies revealed that pain responses are altered by a variety of psychological factors [4, 5, 26, 27], it is possible that pain responses could vary according to general anxiety levels and anxiety specific to pain.

The present study compared the effect of music with non-musical control conditions like news listening and no sound on pain experience while completing a CPT (Hypothesis 1). The 3x3 crossover design was used to better assess the analgesic effects of music. We also explored whether the music intervention was differentially effective based on participants’ general anxiety levels and pain-specific anxiety symptoms (Hypotheses 2 and 3). We followed recommendations from previous CPT pain studies that proposed that pain should be measured in a variety of ways, including pain tolerance time (PT), intensity (PI) and unpleasantness (PU) [15, 28].

## 2. Methods

### Experimental design

This study employed a randomized 3x3 crossover design. Each participant received a sequence of three conditions (control, news, music), and the effects of these three were compared. Each sequence order was determined a priori, and participants were randomly assigned to a particular sequence. Within each sequence group, washout periods were set to 30 minutes; this was done to prevent any carryover effects from one condition to the next. CPT pain variables (PT, PI, PU) were measured after each treatment condition.

### Participants

The Chung-Ang University Institutional Review Board approved this study, and participants provided written informed consents. Participants who exhibited one or more of the following conditions were excluded from the study: diagnosed with an acute or chronic pain-related illness, history of cardiovascular disorders, taking any pain medications, an open sore or cut on either hand, history of hand fracture, or a history of frostbite [29]. We also ensured that no participant experienced adverse medical problems by CPT during oral debriefing. A total of 54 healthy volunteers, 27 men and 27 women, aged 21–35 years, were recruited through poster advertisements on the Chung-Ang University campus noticeboard. Four of 54 participants were excluded from the analysis because they either walked out during the briefing session of the trial, or they appealed the difficulty during the CPT. Demographic and health data prior to the CPT were collected via a questionnaire. We also assessed participants’ experience with music education as an extra-curricular activity to examine whether the effect of music on our main variables of interest would be impacted by prior music experience.

### Procedures and materials

Prior to the CPT, participants were administered two questionnaires to assess anxiety level (16- item Anxiety Sensitive Index; ASI-16) and pain anxiety symptomology (Pain Anxiety Symptom Scale Short Form 20; PASS-20). Participants were informed that the purpose of the experiment was not meant to test their ability to endure ice water and that they should withdraw their hand immediately when the pain became intolerable and to verbally indicate their intention to withdraw. PT was recorded at that moment, and PI and PU were recorded via a visual analog scale (VAS). The VAS comprised a 10-cm line where the left end denotes no pain (0), and the right end denotes worst possible pain (10) [29, 30]. Participants were divided into a “normal group” and “risk group” based on their ASI-16 and PASS-20 scores as a way to factor in the effect of anxiety on music’s analgesic properties.

### CPT

**Fig 1:**
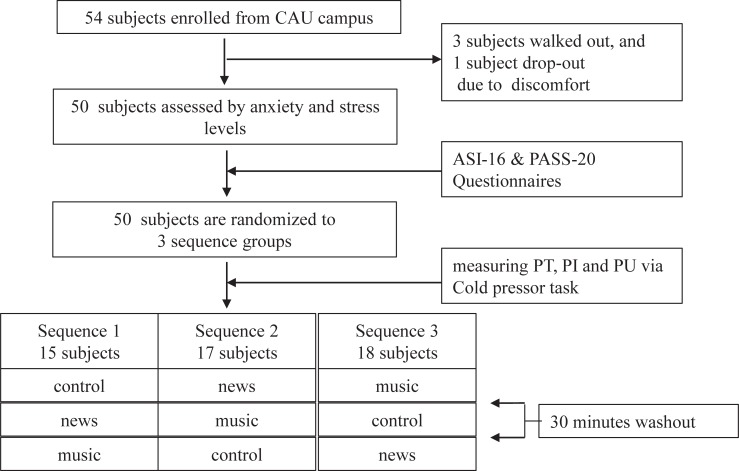
Flow chart depicting participant selection and protocol administration.

The CPT was used to measure vasomotor reactions by immersing the dominant hand into cold water. Two buckets were used—one with tepid water and one with ice water—maintained at 37~40°C and 3~4°C, respectively [15, 31]. The tepid water was provided to help participants return to their normal hand temperature and alleviate their immediate pain. Participants were also instructed to immerse their hand to a depth where the palm touched the bottom of the bucket, while in a standing position, to ensure that immersion was consistent across participants (Fig 1).

## 3. Results

### Demographic characteristics

All statistical analyses were performed using SAS version 9.2 for Windows.

Mean participant age was 25.7 ± 2.9. 24, and 26 participants (52%) were women. Thirty-six participants (72%) had experience with musical instruments. A little over half of the sample (56%) listened to music less than 1 hour per day, and 24% listened between 1 hour and less than 3 hours per day. Most participants (60%) preferred Korean popular music, 32% preferred Western classical music, and one participant preferred Korean traditional music. No participant was currently on any medication (Table 1).

**Table 1:** Participants’ demographic characteristics.

Variable	Sample (N = 50)
Age, years	25.7 (±2.9)
Women, N (%)	26 (52%)
Experience with musical instruments, N (%)	36 (72%)
Average hours listening to music per day, N (%) less than 1 hour1–3 hours3–5 hoursmore than 5 hours	28 (56%) 12 (24%) 4 (8%) 1 (2%)
Music preference rankingKorean popular musicWestern classical musicJazzKorean traditional music	30 (60%) 16 (32%) 3 (6%) 1 (2%)
Medication, N (%)	0 (0%)

Mean (±standard deviation); Frequencies (%)

### Statistical analyses

A separate repeated measures ANOVA was conducted for each pain response variable (PT, PI, and PU), with the three treatments (control, news, and music) as the within-subjects factor (Table 2). Since the SAS mixed procedure program does not provide a typical ANOVA table based on a 3x3 crossover design, Table 2 was re-organized from SAS output to outline the effects assessed as suggested by Jones and Kenward [32].

**Table 2:** ANOVA tables for PT, PI, and PU responses.

Responses		DF	Mean square	F-value	P-value
**Pain Tolerance**					
Between-subjects	Sequence effect Inter-subjects residuals	2 47	64.781 544.915	0.12	0.888
Within-subjects	Period effect Treatment effect Intra-subjects residuals	2 2 96	28.864 417.035 18.507	0.78 22.53	0.461 <0.001
**Pain Intensity**					
Between-subjects	Sequence effect Inter-subjects residuals	2 47	0.958 8.296	0.12	0.891
Within-subjects	Period effect Treatment effect Intra-subjects residuals	2 2 96	1.693 10.800 1.505	1.12 7.18	0.328 0.001
**Pain Unpleasantness**					
Between-subjects	Sequence effect Inter-subjects residuals	2 47	11.894 8.011	1.48	0.237
Within-subjects	Period effect Treatment effect Intra-subjects residuals	2 2 96	1.187 17.524 1.790	1.16 9.79	0.517 <0.001

As shown in Table 2, significant treatment effects were observed for all three pain response variables. Pairwise multiple comparisons tests were conducted to assess which treatment conditions differed from each other across the three pain variables. As shown in Table 3, pain responses during the music condition significantly differed from the news (PT, p < .001; PI, p = .002; PU, p < .001) and control conditions (PT, p < .001; PI, p = .009; PU, p = .022), with PT being higher, and PI and PU being lower. However, pain responses during the news condition did not differ significantly from the control condition for any pain variable (PT, p = .282; PI, p = 1.000; PU, p = .318).

**Table 3:** Pairwise tests for comparing between treatments for each pain variable.

Responses	N	Control	News	Music	P-value*	P-value**	P-value***
Pain Tolerance	50	14.88	16.34	20.45	0.282	< .001	< .001
Pain Intensity	50	6.75	6.65	5.90	1.000	0.002	0.009
Pain Unpleasantness	50	6.84	6.41	5.67	0.318	< .001	0.022

P-value*: listening to news versus control

P-value**: listening to music versus control

P-value***: listening to music versus news

We next investigated the relationship between pain responses and anxiety levels. The ASI-16 (16 item Anxiety Sensitivity Index) is the most widely used measure of the anxiety sensitivity construct. The ASI-16 consists of 16 items used to assess an individual’s fear of anxiety-related symptoms, with responses ranging 0 (very little) to 4 (very much) [33]. Won et al. [34] evaluated the psychometric properties of ASI-16 scores and suggested the possible classification that those with less than 21 on the ASI-16 were categorized into the “normal” group, and those with a score higher than or equal to 21 were categorized into the “risk” group. Table 4 shows results of the Pairwise tests for pain responses during the music, the news, and control condition.

**Table 4:** Pairwise tests for comparing pain responses between treatments for those low and high on the ASI-16.

Responses	n	Control	News	Music	P-value*	P-value**	P-value***
**Pain Tolerance**							
Risk group	12	17.77	20.42	22.84	0.525	0.061	0.834
Normal group	38	14.08	15.10	19.74	0.110	< .001	0.004
**Pain Intensity**							
Risk group	12	5.83	6.00	5.25	1.000	0.291	0.408
Normal group	38	7.03	6.87	6.11	1.000	0.002	0.017
**Pain Unpleasantness**							
Risk group	12	5.92	5.33	4.75	0.735	0.092	0.819
Normal group	38	7.11	6.74	5.95	0.654	0.001	0.030

P-value*: listening to news versus control

P-value**: listening to music versus control

P-value***: listening to music verus news

Pain responses during the music condition for the “normal” group differed significantly from the news (PT, p < .001; PI, p = .002; PU, p < .001) and control conditions (PT, p = .004; PI, p = .017; PU, p = .030) in that PT was higher, and PI and PU were lower. This was not the case for the risk group.

Finally, we investigated the relationship between pain responses and individual differences in pain anxiety symptoms. The PASS-20 (20 item Pain Anxiety Symptoms Scale) is the most widely used measure of pain-related anxiety construct and McCracken and Dhingra [35] developed it, with responses ranging 0 (never) to 5 (always). Abrams et al. [36] evaluated the psychometric properties of PASS-20 scores and suggested the possible classification that those with less than and equal to 25 on the PASS-20 were categorized into the “normal” group, and those with a score higher than 25 were categorized into the “risk” group. Table 5 shows that pain responses during the music condition for the normal group differed significantly from control condition, with PT being higher (p < .001), and PI and PU being lower (p = .001 and p < .001, respectively). However, results of pain responses during the music condition did not differ from the news condition (PT, p < .001; PI, p = .115; PU, p = .180). There were no consistent results for the risk group in pain anxiety symptoms when the music condition was compared the news and control condition.

**Table 5:** Pairwise tests for comparing pain response between treatments for those low and high on the PASS-20.

Responses	n	Control	News	Music	P-value *	P-value **	P-value ***
**Pain Tolerance**							
Risk group	31	16.22	17.35	21.35	1.000	< .001	0.004
Normal group	19	12.92	14.80	19.07	0.259	< .001	0.001
**Pain Intensity**							
Risk group	31	6.48	6.81	6.10	1.000	0.572	0.096
Normal group	19	7.16	6.42	5.58	0.115	< .001	0.115
**Pain Unpleasantness**							
Risk group	31	6.61	6.36	5.65	1.000	0.033	0.226
Normal group	19	7.16	6.47	5.68	0.096	0.001	0.180

P-value*: listening to news versus control

P-value**: listening to music versus control

P-value***: listening to music versus news

## 4. Discussion

The key findings of the present study are as follows: 1) music (without considering participant preferences) led to statistically significant differences in pain responses when compared to the non-musical control conditions. Music appeared to have a specific analgesic effect above and beyond an alternative auditory stimulus. 2) The effect of music on pain responses was only observed for those within a normal range of general and pain-specific anxiety symptomology, suggesting that individuals experiencing heightened levels of anxiety may not benefit as much from the analgesic effects of music

### The unique effects of music

The present study assessed whether music, without considering participants’ preferences, could relieve pain responses to a greater extent than an alternative auditory stimulus (news report) or non-intervening control condition using a 3x3 crossover design. Care was taken in the selection of music to avoid bias and variability in the types of music participants could choose across our intervention conditions. Thus, we made sure to choose music that was familiar but not necessarily the top choice among most of our participants. We chose Arirang music, a traditional genre in Korea.

Prior studies have observed significant differences in pain relief between music and non-music conditions [11, 37, 38]. Differences have also been observed between music and other auditory conditions (i.e., white noise) [17]. However, no prior studies have observed whether the analgesic effect of a non-preferred music genre could be observed. Furthermore, comparing non-preferred music with alternative auditory stimuli (i.e., news) enabled the minimization of a bias toward any auditory stimulus impacting pain responses. Our selection of weather news was specific to the criterion we used for the music selection: participants should be able to recognize the context but not be overly intrigued [13].

Previous studies have revealed that preferred music and/or highly familiar music could be especially distracting during pain perception tasks [15, 18, 39]. Furthermore, participants’ emotional state(s), (e.g., increased positive and decreased negative affect) change based on perceived pain when preferred music is the medium [4, 26]. By using non-preferred music, we were able to reveal a generalized analgesic effect of music on participants’ pain responses, suggesting that any type of music (not just music that is within an individual’s preferred genre) can be effective for reducing negative pain experiences.

### Individual differences in general anxiety level and pain-anxiety symptomology

The present study also addressed whether individual differences in general anxiety levels, as well as pain-specific anxiety, influenced pain responses during a music intervention. Research has observed that mood can affect pain perception/tolerance and vice versa [27, 40–43]. Overall, we observed that music appeared to be more analgesic for those within the normal range of general and pain-specific anxiety. Our results are in keeping with past research observing that pain tolerance can be modified in accordance with one’s mood [4, 5]. More specifically, it appears that the analgesic effects of music are not as prominent among those with high general and pain-specific anxiety. This is consistent with past work showing that music does not have a meaningful effect when anxiety and pain levels are high during pre and post-surgery contexts [12, 13, 44]. Recent study of Fritz et al. [45] also addressed that a pain reducing effect of music agency in combination with physical exercise depended on individual pain sensitivity. Thus, when attempting to utilize music as an adjuvant method for relieving pain, individual traits and mood states should be considered.

## 5. Conclusion

The present study revealed that listening to music (even if the music is not in a preferred genre) is effective for relieving pain. However, this effect is most prominent among individuals who are not experiencing heightened levels of stress and anxiety. Future research should address whether music has a unique or additive effect on alleviating pain in comparison to other intervention methods. Finally, it would be useful to determine whether the analgesic effects of music can be improved for individuals experiencing heightened levels of general or pain-specific anxiety. Continued work in this area has the potential for further advocating the efficacy of non-pharmacological interventions toward improving quality of life and well-being.

## Supporting information

S1 Fig Flow chart depicting participant selection and protocol administration. (TIF)

S1 File 3x3 trial data of 50 subjects. (XLSX)
