# Supplementary figures and images for: The analgesic effect of music on cold pressor pain responses: The influence of anxiety and attitude toward pain
Source: PLoS One. 2018 Aug 6;13(8):e0201897. doi: 10.1371/journal.pone.0201897 (PMC6078312; doi:10.1371/journal.pone.0201897)

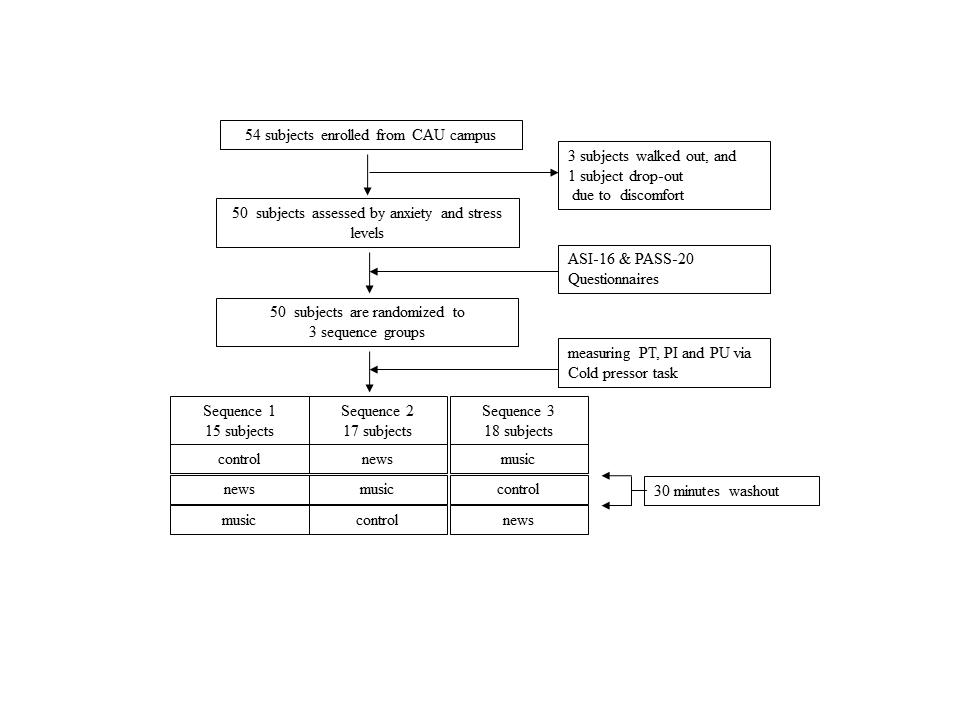

Supplement: S1 Fig — (TIF) [file pone.0201897.s001.tif]
